# Pulmonary Infarction in a Young Man With Situs Inversus Totalis

**DOI:** 10.7759/cureus.71334

**Published:** 2024-10-12

**Authors:** Ancuta-Alina Constantin, Sorin Bivolaru, Radu S Matache, Romina Sima, Florin Dumitru Mihaltan

**Affiliations:** 1 Pulmonology, Institute of Pneumology “Marius Nasta”, Bucharest, ROU; 2 Respiratory Medicine, University of Medicine and Pharmacy “Carol Davila”, Bucharest, ROU; 3 Medical Clinical, Dunarea de Jos University of Galati, Galati, ROU; 4 Thoracic Surgery, Institute of Pneumology “Marius Nasta”, Bucharest, ROU; 5 Obstetrics and Gynecology, University of Medicine and Pharmacy “Carol Davila”, Bucharest, ROU; 6 Pulmonology, University of Medicine and Pharmacy “Carol Davila”, Bucharest, ROU

**Keywords:** lung biopsy, pulmonary infarction, risk factors, situs inversus totalis, thrombophilia

## Abstract

We report the case of a 45-year-old male patient, a smoker, with a known condition of situs inversus totalis (SIT), who was diagnosed with an alveolar consolidation process during a chest imaging examination. Thorough medical investigations, including a surgical lung biopsy, resulted in the diagnosis of pulmonary infarction. The patient's clinical picture began suddenly, with chest pain of a stabbing character on the left side, inspiratory dyspnea, one episode of hemoptysis, fever (40°C), chills, and profuse sweating.

Pulmonary infarction can have many different causes and determining the underlying etiology is frequently a considerable challenge, particularly given the urgency imposed by the severity of the condition. The association of pulmonary infarction in a patient with SIT is particularly noteworthy, as each of these conditions represents distinct pathological entities, with their overlap addressed in only a few cases in the literature. A rare genetic predisposition, possibly a fairly ordinary pairing, or even incidental coexistence, are some of the speculations discussed in this case presentation.

We emphasize that pulmonary consolidation requires a comprehensive diagnostic approach due to its broad differential diagnosis. This highlights the critical importance of surgical lung biopsy and histopathological analysis in securing a precise and accurate diagnosis.

## Introduction

Situs inversus totalis is a rare congenital anomaly marked by the complete transposition of thoraco-abdominal organs. It occurs in approximately 1 in 10,000 births, with no significant racial differences and a slight male predominance (1.5:1) [1, 2]. While the precise etiology is not well understood, potential contributing factors include genetic abnormalities, maternal diabetes, and a family history of heart disease. Although SIT can occur as an isolated condition, commonly associated comorbidities include primary ciliary dyskinesia, Kartagener syndrome, congenital cardiovascular defects, and biliary atresia, which add to the complexity [1-4].

## Case presentation

**Figure 1:**
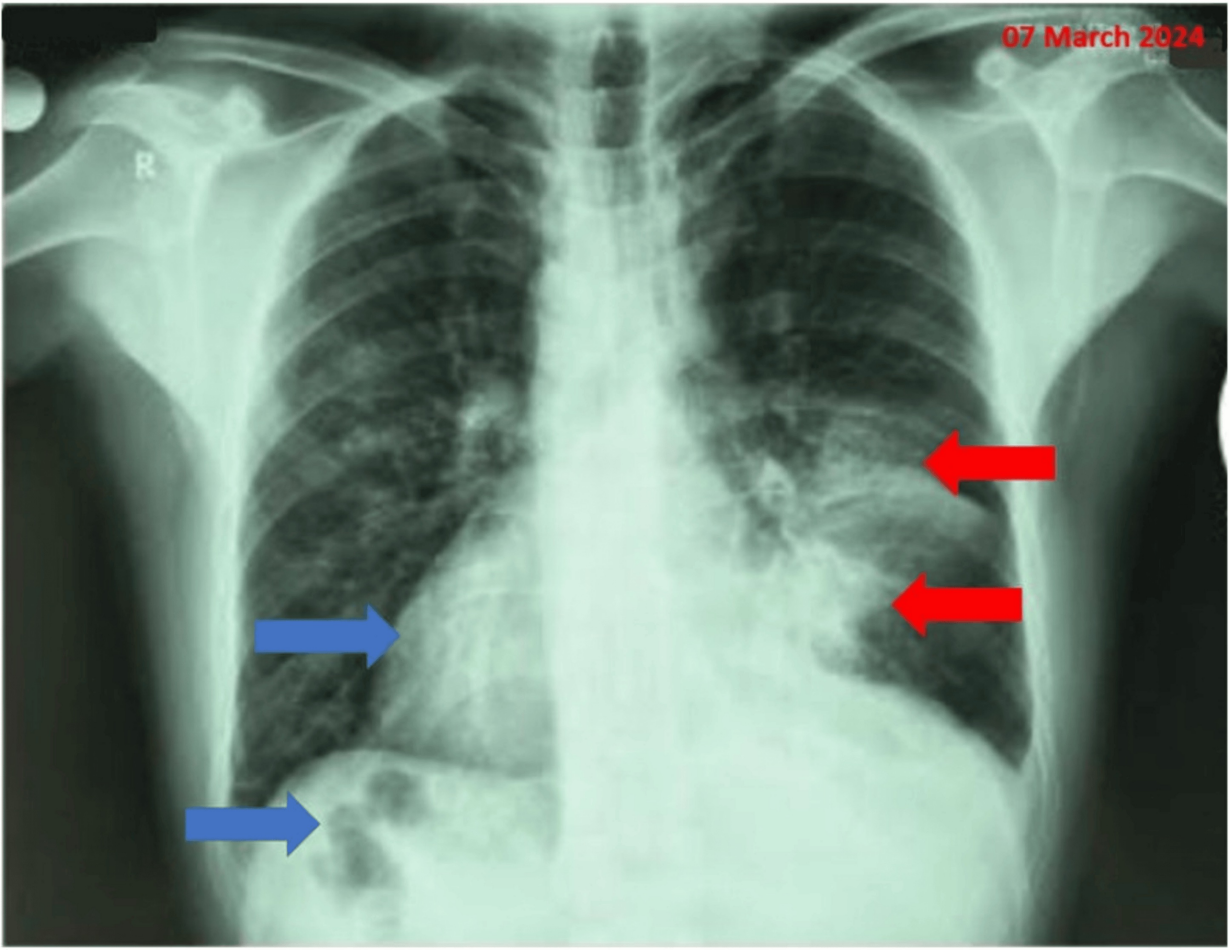
Chest X-ray revealing malposition (mirrored anatomy) characteristic of SIT

We present the case of a 45-year-old male patient, a current smoker with a history of 20 pack-years, and no professional or household exposure to respiratory pollutants. He was referred to our medical department for in-depth investigations of an alveolar consolidation syndrome, identified radiologically in the middle third of the left lung field, following preliminary evaluations performed in a local medical center. Anamnestically, the patient denies any chronic home treatment, and no somatic comorbidities were noted, except for a diagnosis of situs inversus totalis in childhood, which did not require periodic follow-up or additional hospitalizations. Retrospectively, we find that in March 2024, he visited a local healthcare facility, reporting left-sided pleuritic chest pain, inspiratory dyspnea, a single episode of hemoptysis, high fever, chills, and sweating. This symptomatology was labeled as infectious. A chest X-ray was performed (Figure 1), revealing that the heart is located on the right side (dextrocardia) and the stomach bubble is positioned beneath the right hemidiaphragm. Additionally, there is a homogeneous opacity of moderate intensity, systematically situated parahilar on the left side, within the middle third of the left lung field, accompanied by obliteration of the ipsilateral costodiaphragmatic recess (red arrow). To achieve a more precise diagnostic evaluation of these radiological findings, a chest computed tomography (CT) scan was recommended. Meanwhile, urgent antibiotic therapy was initiated, with a firm recommendation to pursue further respiratory investigations.

**Figure 2:**
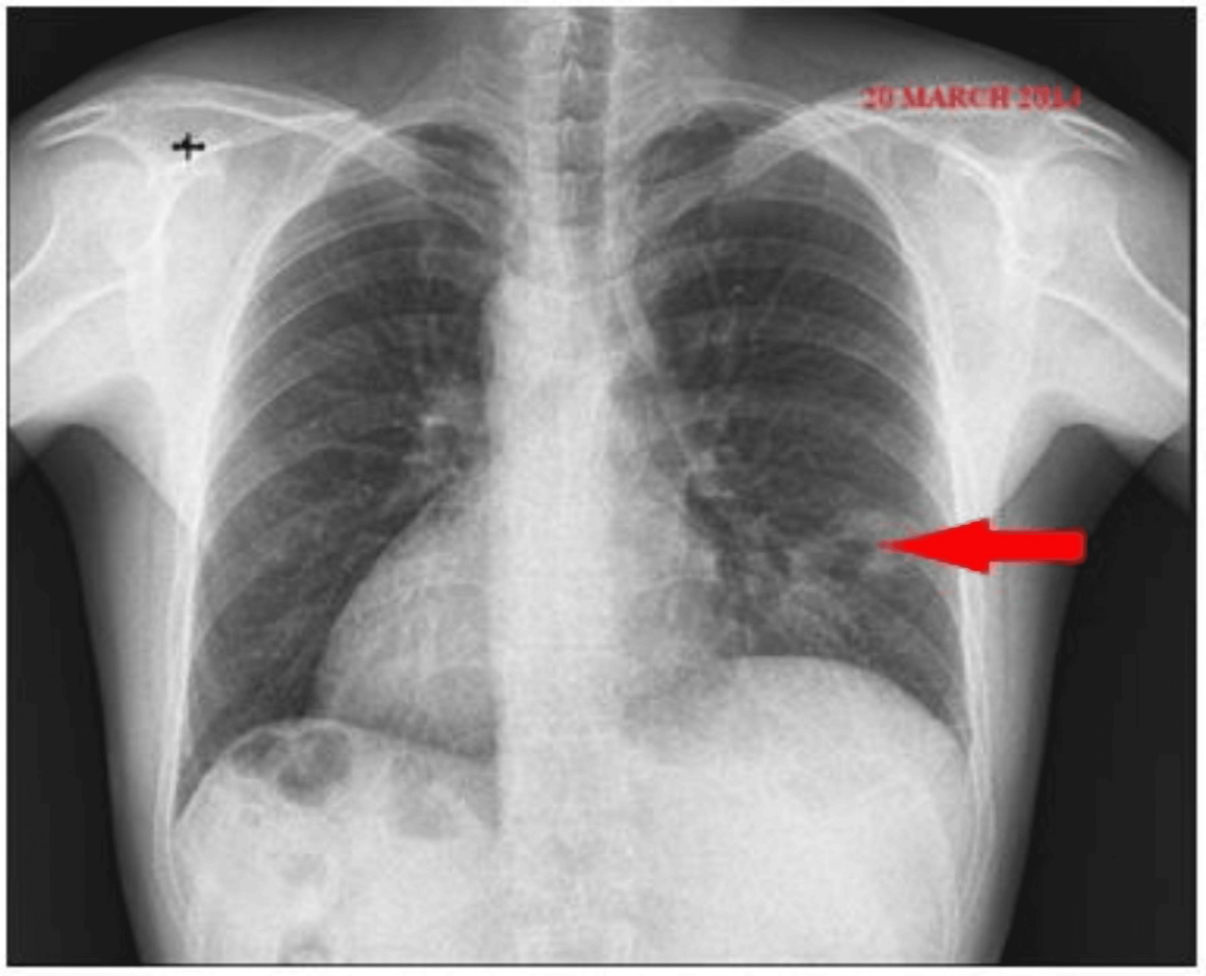
Chest X-ray showing persistence of the left paracardiac pulmonary infiltrate

Due to the limited clinical and radiological response to the antibiotic and symptomatic treatment, as observed in the second X-ray (Figure 2), a decision was made to proceed with advanced imaging evaluation through a chest CT scan.

**Figure 3:**
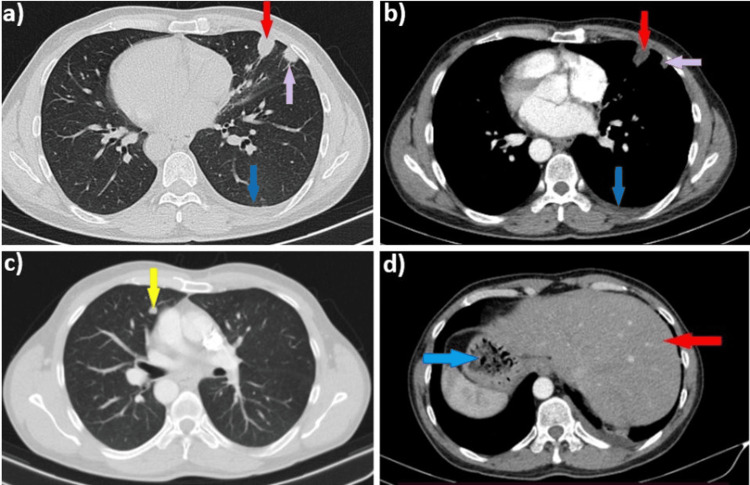
Chest CT scan showing subpleural nodular densifications, pleural effusion, and a left paracardiac micronodule

The chest CT scan revealed a small nodular pulmonary consolidation with an air bronchogram, located subpleural on the right pulmonary, based on the chest wall, situated in the left pulmonary field due to his condition (Figures 3a, 3b - red arrow). Additionally, another discrete pulmonary consolidation was noted, adjacent to the aforementioned consolidation (Figure 3a, 3b - purple arrow), as well as a small oval pulmonary micronodule with a heterogeneous structure, located paracardially on the contralateral side (Figure 3c - yellow arrow). The scan also showed left hilar lymphadenopathy (12 mm) and fluid accumulation in the posterior left costophrenic sinus (Figures 3a, 3b - blue arrow). Additionally, as previously noted, the liver is observed on the left side (Figure 3d - red arrow), while the gastric fornix is positioned on the right (Figure 3d - blue arrow), which are hallmark features of SIT.

After his admission to our clinic, we reviewed the previous investigations and determined that additional pulmonary function tests were necessary. These showed normal values of ventilatory volumes and flow rates, with a slight decrease in the diffusing capacity of the lung for carbon monoxide (DLCO), with an estimated value of 64.2% of the predicted values. Additionally, the patient underwent a fiberoptic bronchoscopy, which revealed normal laryngeal dynamics and no apparent proliferative elements or active lesions in the examined mucosal tissue. Bronchoalveolar lavage (BAL) was performed at the level of the left main bronchus (120 ml instilled, moderate recovery) for cytodiagnosis. The BAL fluid was also evaluated for bacteriological exams and yielded no pathogenic microorganisms. Ziehl-Neelsen staining of the lavage fluid revealed no acid-fast bacilli.

Blood tests indicated a nonspecific inflammatory syndrome [erythrocyte sedimentation rate (ESR) = 51 mm/h, c-reactive protein (CRP) value was slightly elevated compared to the normal value -5.2 mg/L, versus 5 mg/L, but without infection markers, without leukocytosis, and with normal serum procalcitonin levels], mild iron deficiency anemia (Fe = 56 ug/dL, Hb = 11.40 g/dL), elevated D-dimers (twice the normal value), mild cytolysis [alanine transaminase (ALT) = 93 U/L], and moderate thrombocytosis. Serological testing for connective tissue diseases (CTD) - rheumatoid factor (RF), and antinuclear antibodies (ANA) screen was negative (Table 1).

**Table 1:** Laboratory blood test AST: aspartate transferase, ALT: alanine transaminase, CRP: c-reactive protein, ESR: erythrocyte sedimentation rate, HBs Ag: hepatitis B surface antigen, HIV: human immunodeficiency virus, ANA: Antinuclear Antibody, dsDNA: double-stranded DNA.

Laboratory test	Hospital admission	Normal range
Hemoglobin (g/dL)	11.40	13.5-16.9
Hematocrit (%)	35.50	40-49.4
Total leukocyte count (10^3^/µL)	8,120	3,90-10,90
Lymphocyte %()	24.50	19.1-47.9
Monocyte (%)	6.90	5.2-15.2
Eosinophils (%)	1.5	0.6-7.6
Basophil (%)	0.50	0.1-1.2
Platelets (10^3^/µL)	468.00	166-368
Creatinine (mg/dL)	0.59	0.67-1.17
Urea (mg/dL)	30	17-43
AST (U/L)	47	0-50
ALT (U/L)	93	0-50
CRP (mg/L)	5.2	0-5
ESR (mm/h)	51	2-15
Procalcitonin (ng/mL)	0.05	0.0-0.5
Serum Iron (µg/dL)	56	70-180
D-Dimers (ng/mL)	505	0-243
HBs Ag	Negative	Negative
Hepatitis C Ab	Negative	Negative
HIV	Negative	Negative
Rheumatoid factor	<10	0-10
ANA screen (AU/mL)	6.96	0-40
Anti dsDNA antibodies (AU/mL)	0.50	0-30

**Figure 4:**
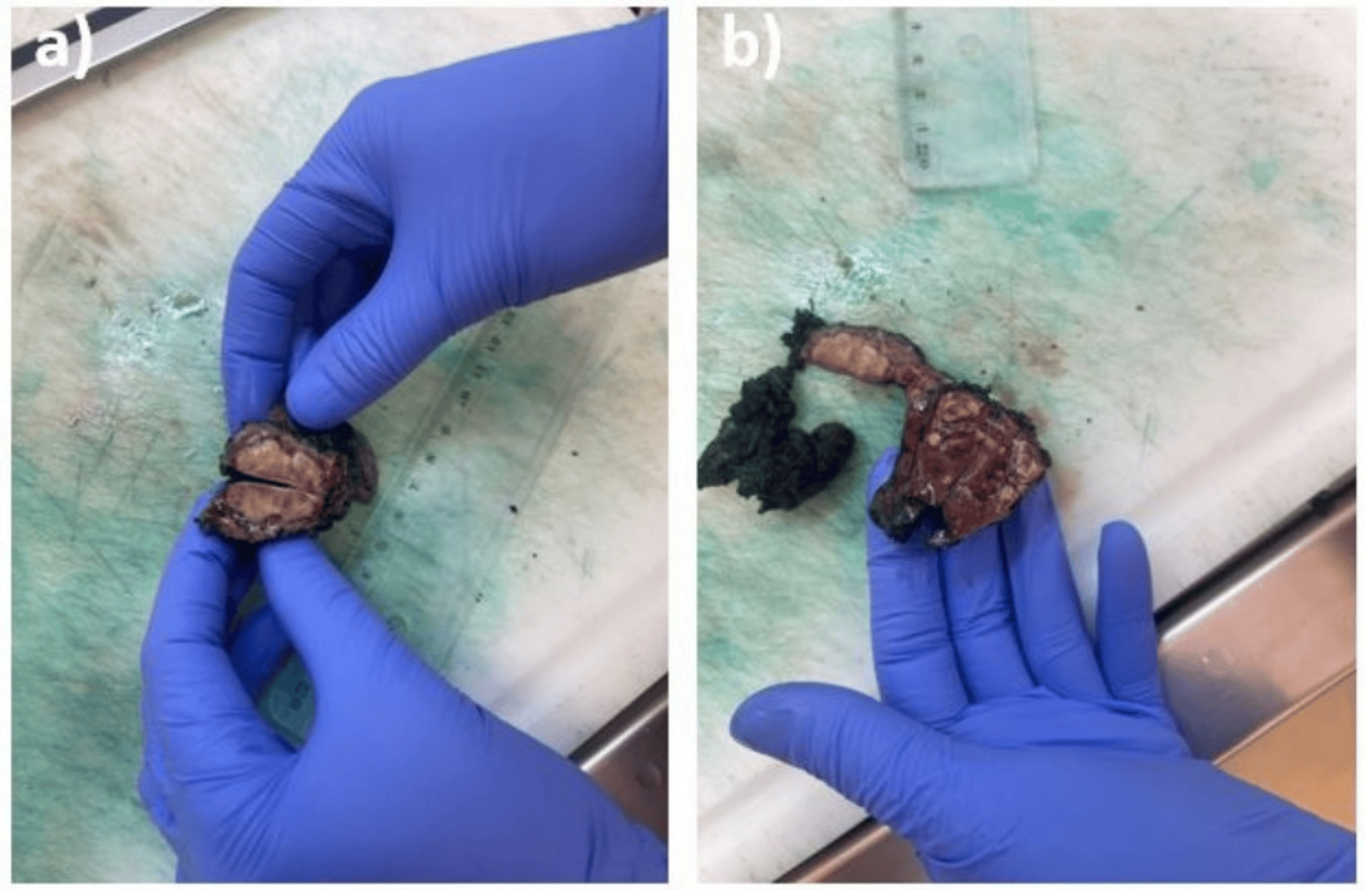
Bioptic samples surgically resected

Given that the follow-up radiological assessment did not demonstrate complete regression of the pulmonary infiltrate despite the administration of broad-spectrum antibiotics for approximately three weeks, and considering the patient's relatively modest clinical condition, a surgical lung biopsy was recommended to ensure diagnostic accuracy. Therefore, the patient was referred to the thoracic surgery clinic for a lung biopsy to obtain a definitive diagnosis and subsequently determine the appropriate treatment approach. Under general anesthesia with selective intubation, a left thoracotomy was performed with atypical resection of the middle lobe. The intraoperative examination described the absence of tumor characteristics and indicated pulmonary infarction with multiple vascular thromboses (Figures 4a, 4b). The histopathological examination described a pleuropulmonary fragment measuring 8 x 4.5 x 1.8 cm. On-section, four nodules were identified with variable dimensions ranging from 5 x 4 x 4 mm to 24 x 11 x 20 mm. The nodules were white-yellowish, with slight anthracotic staining, increased consistency, and elastic texture. The remaining lung parenchyma appeared congested.

**Figure 5:**
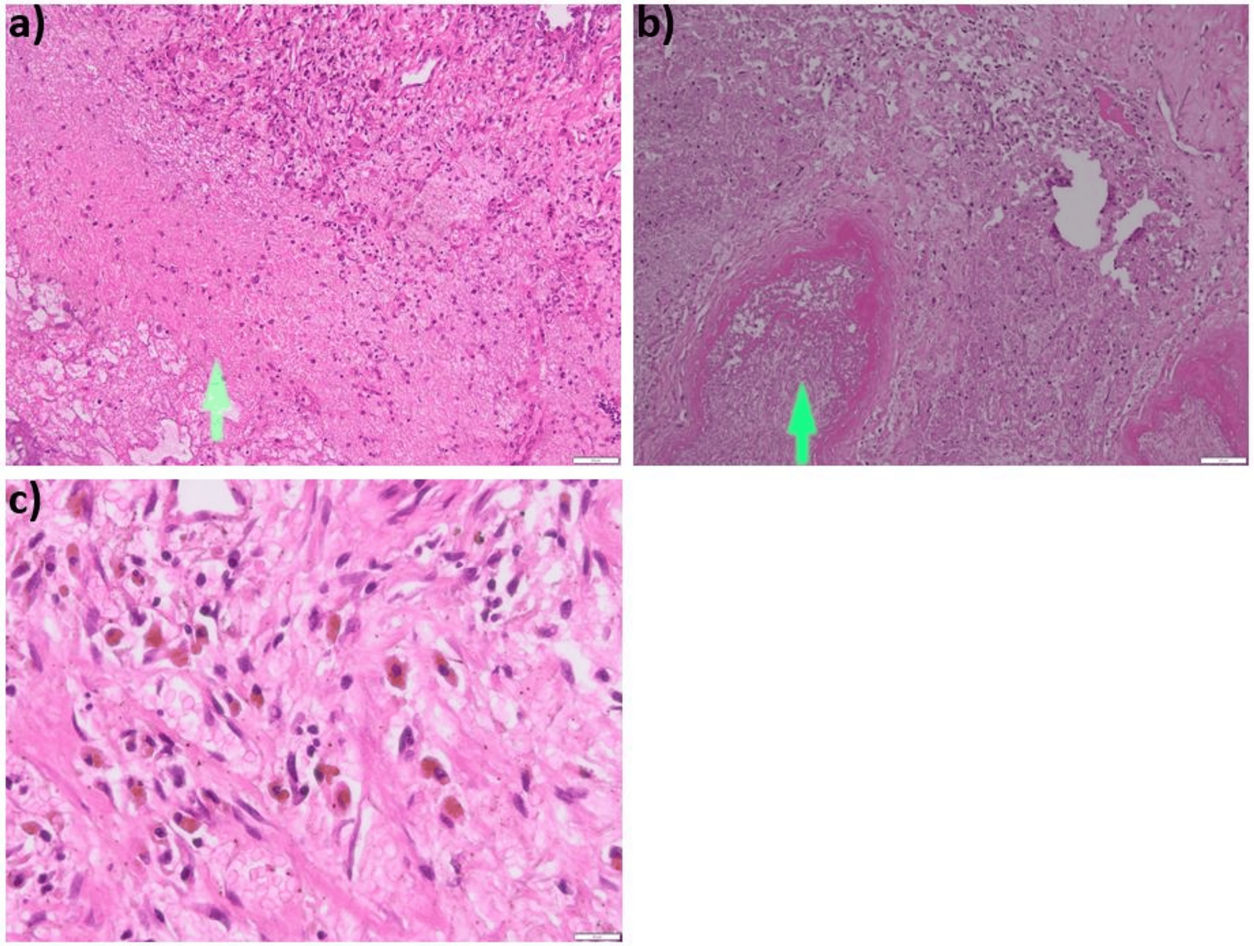
Histopathology samples indicating pulmonary infarction a) (10x). Standard hematoxylin and eosin staining. The area of hemorrhagic necrosis is observed (the interalveolar septa are destroyed, only shadows of septa can be seen, the lung parenchyma is replaced by fibrin and red blood cells); the necrotic area is bordered by granulation tissue. b) (10x) Recent thrombus - the vascular lumen is filled with fibrin and red blood cells. c) (10x) Masson's trichrome staining revealed collagen deposits, stained blue, at the periphery of the infarct (the infarct area is stained red, located on the left side)

Histopathological analysis revealed extensive coagulative necrosis, with visible red blood cells (most undergoing lysis), fibrin, and rare neutrophils and lymphocytes. The necrotic area was surrounded by remodeled lung parenchyma showing significant fibroblastic and fibrocystic proliferation, interstitial collagen deposition, and residual alveoli with hypertrophied epithelium and focal squamous metaplasia. Endoalveolar findings included red blood cells, fibrin, and siderophages. The arterioles in this area had thickened walls and contained organized thrombi, some of which were re-canalized. Additionally, several fibrogranulative buds of organized pneumonia (OP) type and a foreign body giant cell reaction were observed. Congo Red staining did not reveal amyloid deposits. Overall, the histopathological findings are suggestive of an infarction diagnosis (Figure 5).

From this juncture onward, the differential diagnosis has been progressively expanded to include inflammatory-infectious, neoplastic, and thrombophilic conditions, as well as rare disorders such as amyloidosis.

Following the results of the histopathological examination, a hematology consultation was conducted and further investigations were recommended to rule out thrombophilia or other underlying causes. The recommended tests targeted a wide spectrum, including protein C, protein S, antithrombin III, lupus anticoagulant, factor V Leiden mutation, prothrombin factor II mutation, PAI-1 gene, factor XIII mutation, methylenetetrahydrofolate reductase (MTHFR) gene, and homocysteine. It was also recommended to continue anticoagulant treatment with low molecular weight heparin (LMWH) and to undergo a cardiological evaluation for the initiation of oral anticoagulant therapy with Eliquis 5 mg, twice daily for six months. After hematological evaluation, a positive lupus anticoagulant was detected (1.46 versus negative). Additionally, the PAI-1 4G and ACE D alleles were found in heterozygosity in the analyzed sample. As a result, the patient will need to remain on anticoagulation therapy indefinitely (Table 2).

**Table 2:** Thrombophilia laboratory tests

Laboratory test	Value	Normal range
Lupus Anticoagulant	1.46 Positive	Negative
Protein C (%)	102%	70-130
Protein S free (%)	98%	60-140
Antithrombin III (%)	115%	80-120
Factor V Leiden	Normal	Normal

**Figure 6:**
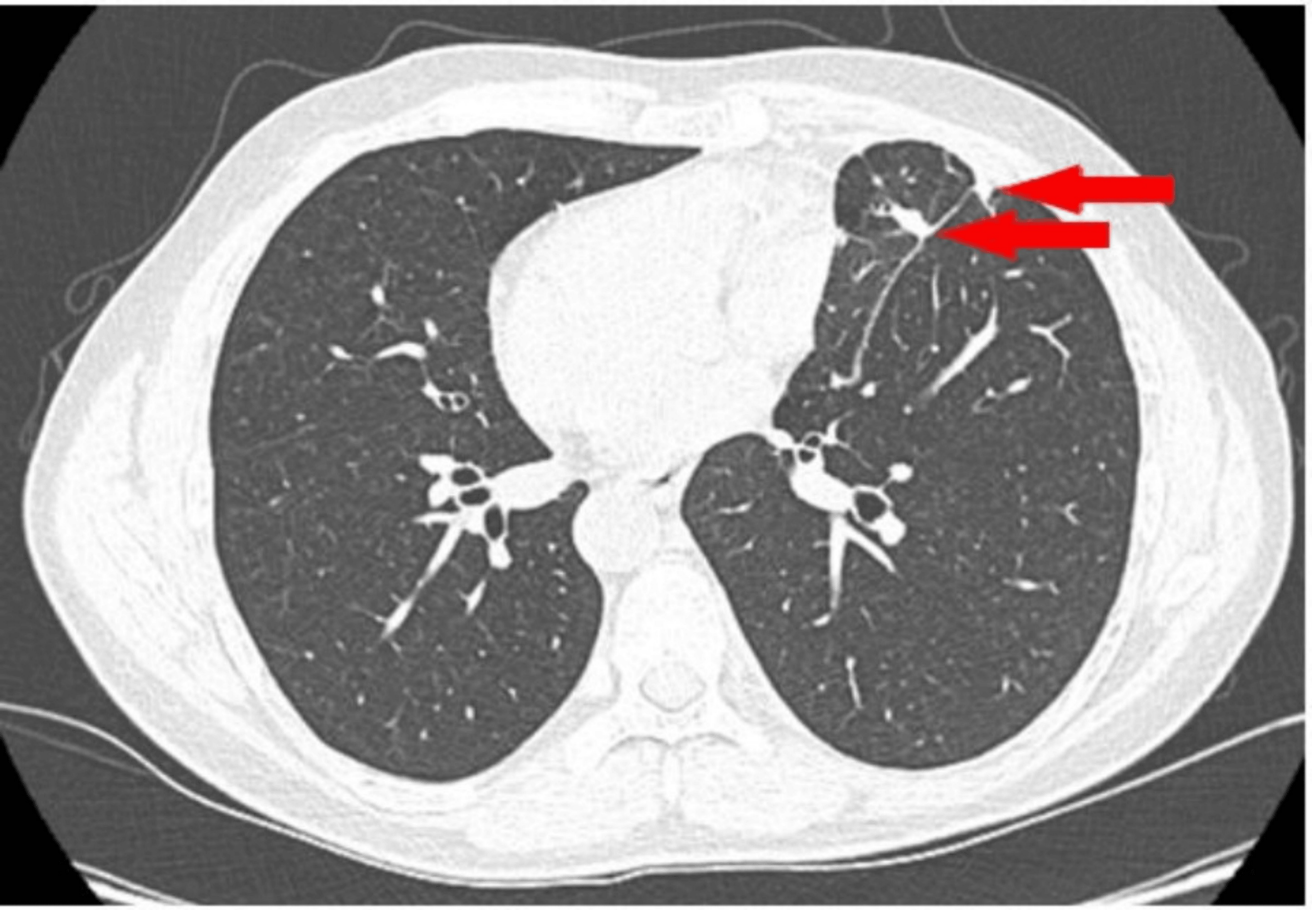
Cest CT postoperative residual changes

Imaging reevaluation after two months shows evidence of favorable progression, with no recurrence of the previously mentioned pleural fluid accumulation. The right pulmonary micronodule remains stable, with only minimal left residual postoperative fibrous lesions present (Figure 6), and the postoperative course was uneventful.

Subsequently, the cardiology consultation, including echocardiography, did not reveal any significant abnormalities. The echocardiogram demonstrated a normal left ventricular cavity size with an ejection fraction of 63% and cardiac cavities of normal dimensions, with no evidence of wall motion abnormalities.

## Discussion

The case presents two significant pathologies occurring simultaneously and seemingly not incidentally in this patient, with the first pathological condition potentially predisposing to the occurrence of a thromboembolic state. As in the present case, an additional challenge for patients with SIT who require thoracic surgery arises from the fact that these procedures can carry specific perioperative risks for both the surgical and anesthesia teams, as reported in other publications in the literature [3, 5, 6].

Our patient was not assessed for any genetic disorders, as he was asymptomatic regarding his situs inversus condition, and the preoperative assessments confirmed SIT with no additional anomalies. Although most patients with SIT are asymptomatic, the emergence of acute pathology can markedly influence their prognosis. In our patient, the characteristic symptoms of pulmonary infarction, including pleuritic chest pain, hemoptysis (30%), and episodes of sub-febrile or febrile temperatures [7], were initially misattributed to an inflammatory-infectious origin. This misinterpretation led to a delay in the patient's referral to our clinic. Once there, in the absence of significant clinical and imaging improvement despite antibiotic therapy, further diagnostic investigations were undertaken, ultimately resulting in an accurate diagnosis.

Given the peripheral position of the lesion, a transthoracic biopsy would certainly have been preferable; however, the diagnostic process was considered in the long term, considering the cumulative factors assessed by the medical team. The radiologist's description of the air bronchogram is a marker of potential risks for hemothorax and pneumothorax in the case of a lesion. Additionally, the small size of the left subpleural nodular lesions rather supports refraining from a transthoracic biopsy, as the risk of pneumothorax (PTX) is present. In the event of a possible neoplastic context, although transcutaneous biopsy would have provided arguments in establishing the histopathological substrate of the biopsy material, the amount of tissue collected would have been minimal, which could have hindered subsequent immunohistochemistry testing. In the case of confirming a neoplastic lesion through transthoracic biopsy, the initial diagnostic premise would have led to surgery anyway. Therefore, it was decided to prioritize surgical intervention for both diagnostic and therapeutic purposes, with the patient's consent, after explaining the diagnostic course.

Although positron emission tomography (PET)-CT evaluation would have added diagnostic value, it is burdened by false-positive results that can occur in acute inflammation or infectious conditions. In this case, the diagnostic benefit was somewhat limited, as both neoplasia and infection, the two major diagnostic hypotheses, can show increased uptake. In the end, the role of biopsy remains crucial, so if the lesion had persisted, this course of action would most likely have been taken at this point anyway [8].

Since the diagnosis of mixed hereditary and acquired thrombophilia was established, along with a history of pulmonary infarction in a relatively young patient, elevated PAI-1 levels combined with AC polymorphism are associated with an increased risk of pulmonary embolism due to their effects on the renin-angiotensin-aldosterone system, which regulates blood pressure and vascular homeostasis. Evidence is limited, but some studies suggest a significantly higher risk of pulmonary embolism (PE) [9]. The prevalence of thrombosis in patients with lupus anticoagulants ranges from 24% to 36%, with deep vein thrombosis and pulmonary embolism being the most common. Given the patient's history and the high suspicion of lupus, lifelong oral anticoagulant therapy remains recommended. Selective pulmonary arteriography is considered the gold standard for diagnosing pulmonary embolism, as it precisely delineates the location and extent of thromboembolic lesions. The integration of right heart catheterization data provides detailed insights into the magnitude of hemodynamic alterations. Additionally, the combined use of perfusion and ventilation scintigraphy enhances the diagnostic specificity of the evaluation [7]. An important avenue for future research emerges from the association between situs SIT and thrombophilia. Literature on this topic is limited, suggesting either the rarity of these cases or potential underreporting. The proposed hypotheses refer to structural or functional abnormalities associated with SIT, which could impact normal hemodynamics and alter the risk of thrombus formation. Moreover, the potential genetic predisposition linking SIT and thrombophilic disorders has been discussed, particularly since both conditions may coexist within certain rare genetic syndromes, such as Kartagener syndrome. This syndrome, characterized by situs inversus, can include pulmonary manifestations that are prone to inflammation and infections. What is certain is that situs inversus totalis remains a rare abnormality with an uncertain pathophysiology [10].

Although situs inversus totalis is primarily linked to an autosomal recessive pattern of transmission [11], Arash et al. note that environmental factors may also contribute to its development. Key risk factors include a family history of congenital heart defects, a family history of non-cardiac anomalies, inadequate management of maternal diabetes during pregnancy, use of antitussives, paternal smoking, and low socio-economic status [12]. Genetic factors implicated in these defects include the lefty genes, nodal genes, ZIC3, ACVR2B, DNAH11, and Pitx2 genes. Additionally, serotonin (5HT) has been identified as a critical regulator of laterality [11, 13, 14].

## Conclusions

Following a thorough review of the literature, no similar cases have been reported, to the best of our knowledge. In this case, the imaging aspect is atypical for pulmonary infarction, presenting as a round-oval opacity and not conforming to the classic presentation of triangular condensation with the base at the pleura and the apex at the hilum. Thus, the pulmonary nodule represents a pathological entity that continues to yield diagnostic surprises, as its etiological polymorphism can unexpectedly challenge clinical assessment. Therefore, in cases of peripheral lung nodules located in subpleural regions, the potential for pulmonary infarctions should be considered, even when there is no evidence of venous thromboembolism. Pulmonary infarctions result from pulmonary thromboembolism, heart failure, vasculitis, infection, and malignancy. Future research should explore the potential genetic and pathophysiological connections between SIT and thromboembolic disorders, and work towards developing specific management guidelines for patients with these coexisting conditions. In this context, additional cohort studies and genetic analyses are necessary to investigate this potential association and to assess whether patients with SIT require specialized monitoring for thromboembolic disorders. Furthermore, the case is instructive and underscores the importance of an interdisciplinary approach; such collaboration is crucial for comprehensive care, especially in complex cases involving rare congenital anomalies like SIT. 
